# Supplementary material for: The Ndc80 complex bridges two Dam1 complex rings
Source: eLife. 2017 Feb 13;6:e21069. doi: 10.7554/eLife.21069 (PMC5354518; doi:10.7554/eLife.21069)
Supplement: Supplementary file 1. — DOI: http://dx.doi.org/10.7554/eLife.21069.029 [file elife-21069-supp1.docx]

A. Pairwise statistical comparisons for Figure 1D

| Sample 1 (proteins on microtubules) | Sample 2 (proteins on microtubules) | p-value |
| --- | --- | --- |
| wt Ndc80c | wt Ndc80c + Dam1c mock | <0.001 |
| wt Ndc80c | Mutant A Ndc80c | 0.17 |
| wt Ndc80c | Mutant B Ndc80c | 0.089 |
| wt Ndc80c | Mutant C Ndc80c | 0.089 |
| Mutant A Ndc80c | Mutant A Ndc80c + Dam1c mock | <0.001 |
| Mutant B Ndc80c | Mutant B Ndc80c + Dam1c mock | 0.0016 |
| Mutant C Ndc80c | Mutant C Ndc80c + Dam1c mock | <0.001 |

B. Pairwise statistical comparisons for Figure 1 – figure supplement 3C

| Sample 1 (proteins on microtubules) | Sample 2 (proteins on microtubules) | p-value |
| --- | --- | --- |
| wt Ndc80c | Ndc80c ins 219 | 0.04 |
| wt Ndc80c | Ndc80c ins 652 | 0.04 |
| wt Ndc80c + Dam1c mock | Ndc80c ins 219 + Dam1c mock | 0.20 |
| wt Ndc80c + Dam1c mock | Ndc80c ins 652 + Dam1c mock | 0.074 |

C. Pairwise statistical comparisons for Figure 2

| Sample 1 (proteins on microtubules) | Sample 2 (proteins on microtubules) | p-value |
| --- | --- | --- |
| wt Ndc80c | wt Ndc80c + Dam1c phos | 0.48 |
| wt Ndc80c + Dam1c mock | wt Ndc80c + Dam1c phos | <0.001 |
| wt Ndc80c + Dam1c mock | wt Ndc80c + Dam1c 6A phos | 0.48 |
| wt Ndc80c + Dam1c phos | wt Ndc80c + Dam1c 6A phos | <0.001 |
| wt Ndc80c | wt Ndc80c + Dam1c A phos | <0.001 |
| wt Ndc80c | wt Ndc80c + Dam1c B phos | <0.001 |
| wt Ndc80c | wt Ndc80c + Dam1c C phos | <0.001 |
| wt Ndc80c | wt Ndc80c + Dam1c B,C phos | <0.001 |
| wt Ndc80c | wt Ndc80c + Dam1c A,B phos | 0.29 |
| wt Ndc80c | wt Ndc80c + Dam1c A,C phos | 0.29 |
| wt Ndc80c + Dam1c mock | wt Ndc80c + Dam1c A phos | <0.001 |
| wt Ndc80c + Dam1c mock | wt Ndc80c + Dam1c B phos | <0.001 |
| wt Ndc80c + Dam1c mock | wt Ndc80c + Dam1c C phos | <0.001 |
| wt Ndc80c + Dam1c mock | wt Ndc80c + Dam1c B,C phos | <0.001 |
| wt Ndc80c + Dam1c mock | wt Ndc80c + Dam1c A,B phos | <0.001 |
| wt Ndc80c + Dam1c mock | wt Ndc80c + Dam1c A,C phos | <0.001 |
| wt Ndc80c + Dam1c A phos | wt Ndc80c + Dam1c A,B phos | <0.001 |
| wt Ndc80c + Dam1c A phos | wt Ndc80c + Dam1c A,C phos | <0.001 |
| wt Ndc80c + Dam1c B phos | wt Ndc80c + Dam1c A,B phos | <0.001 |
| wt Ndc80c + Dam1c C phos | wt Ndc80c + Dam1c A,C phos | 0.0022 |
| wt Ndc80c + Dam1c B phos | wt Ndc80c + Dam1c B,C phos | 0.074 |
| wt Ndc80c + Dam1c C phos | wt Ndc80c + Dam1c B,C phos | 0.33 |
| wt Ndc80c + Dam1c B phos | mut C Ndc80c + Dam1c B phos | 0.26 |
| wt Ndc80c + Dam1c C phos | mut B Ndc80c + Dam1c C phos | 0.026 |
| wt Ndc80c + Dam1c B phos | mut A Ndc80c + Dam1c B phos | <0.001 |
| wt Ndc80c + Dam1c A phos | mut B Ndc80c + Dam1c A phos | 0.0054 |
| wt Ndc80c + Dam1c C phos | mut A Ndc80c + Dam1c C phos | 0.13 |
| wt Ndc80c + Dam1c A phos | mut C Ndc80c + Dam1c A phos | <0.001 |
| mut C Ndc80c + Dam1c mock | mut C Ndc80c + Dam1c B phos | 0.12 |
| mut B Ndc80c + Dam1c mock | mut B Ndc80c + Dam1c C phos | 0.48 |
| mut A Ndc80c + Dam1c mock | mut A Ndc80c + Dam1c B phos | 0.0016 |
| mut B Ndc80c + Dam1c mock | mut B Ndc80c + Dam1c A phos | 0.0011 |
| mut A Ndc80c + Dam1c mock | mut A Ndc80c + Dam1c C phos | 0.0094 |
| mut C Ndc80c + Dam1c mock | mut C Ndc80c + Dam1c A phos | <0.001 |
| wt Ndc80c + Dam1c B,C phos | mut C Ndc80c + Dam1c B phos | 0.26 |
| wt Ndc80c + Dam1c B,C phos | mut B Ndc80c + Dam1c C phos | 0.048 |
| wt Ndc80c + Dam1c A,B phos | mut A Ndc80c + Dam1c B phos | 0.13 |
| wt Ndc80c + Dam1c A,B phos | mut B Ndc80c + Dam1c A phos | 0.48 |
| wt Ndc80c + Dam1c A,C phos | mut A Ndc80c + Dam1c C phos | 0.23 |
| wt Ndc80c + Dam1c A,C phos | mut C Ndc80c + Dam1c A phos | 0.48 |

D. Pairwise statistical comparisons for Figure 2 – figure supplement 2

| Sample 1 (proteins on microtubules) | Sample 2 (proteins on microtubules) | p-value |
| --- | --- | --- |
| wt Ndc80c | wt Ndc80c + Dam1c 6A mock | <0.001 |
| wt Ndc80c | wt Ndc80c + Dam1c site A Ala mock | <0.001 |
| wt Ndc80c | wt Ndc80c + Dam1c site B Ala mock | <0.001 |
| wt Ndc80c | wt Ndc80c + Dam1c site C Ala mock | <0.001 |
| wt Ndc80c | wt Ndc80c + Dam1c site B,C Ala mock | <0.001 |
| wt Ndc80c | wt Ndc80c + Dam1c site A,B Ala mock | <0.001 |
| wt Ndc80c | wt Ndc80c + Dam1c site A,C Ala mock | <0.001 |
| wt Ndc80c + Dam1c mock | wt Ndc80c + Dam1c 6A mock | 0.074 |
| wt Ndc80c + Dam1c mock | wt Ndc80c + Dam1c site A Ala mock | 0.074 |
| wt Ndc80c + Dam1c mock | wt Ndc80c + Dam1c site B Ala mock | 0.48 |
| wt Ndc80c + Dam1c mock | wt Ndc80c + Dam1c site C Ala mock | 0.040 |
| wt Ndc80c + Dam1c mock | wt Ndc80c + Dam1c site B,C Ala mock | 0.26 |
| wt Ndc80c + Dam1 mock | wt Ndc80c + Dam1c site A,B Ala mock | 0.48 |
| wt Ndc80c + Dam1 mock | wt Ndc80c + Dam1c site A,C Ala mock | 0.0734 |

| Sample 1 | Sample 2 | p-value |
| --- | --- | --- |
| no Ndc80 complex | wt Ndc80 complex | <0.001 |
| no Ndc80 complex | Ndc80^10hep^ complex | <0.001 |
| no Ndc80 complex | Mutant A Ndc80 complex | 0.48 |
| no Ndc80 complex | Mutant B Ndc80 complex | 0.44 |
| no Ndc80 complex | Mutant C Ndc80 complex | 0.44 |
| wt Ndc80 complex | Ndc80^10hep^ complex | 0.016 |
| wt Ndc80 complex | Mutant A Ndc80 complex | <0.001 |
| wt Ndc80 complex | Mutant B Ndc80 complex | <0.001 |
| wt Ndc80 complex | Mutant C Ndc80 complex | <0.001 |
| Ndc80^10hep^ complex | Mutant A Ndc80 complex | <0.001 |
| Ndc80^10hep^ complex | Mutant B Ndc80 complex | <0.001 |
| Ndc80^10hep^ complex | Mutant C Ndc80 complex | <0.001 |
| Mutant A Ndc80 complex | Mutant B Ndc80 complex | 0.44 |
| Mutant A Ndc80 complex | Mutant C Ndc80 complex | 0.44 |
| Mutant B Ndc80 complex | Mutant C Ndc80 complex | 0.48 |

E. Pairwise statistical comparisons for Figure 3 and Figure 3 – figure supplement 2

F. Pairwise statistical comparisons for Figure 4

| Sample 1 (proteins on microtubules) | Sample 1 (proteins on microtubules) | p-value |
| --- | --- | --- |
| Ndc80c^10hep^ | Ndc80^10hep^ + Dam1c mock | <0.001 |
| wt Ndc80c | Ndc80^10hep^ | 0.29 |
| wt Ndc80c + Dam1c mock | Ndc80^10hep^ + Dam1c mock | 0.36 |

G. Pairwise statistical comparisons for Figure 6C

| Sample 1 (Ndc80 mutation) | Sample 2 | p-value |
| --- | --- | --- |
| Ndc80 depleted (null) | Wild-type Ndc80 | <0.001 |
| Ndc80 depleted | Region A^Ndc80p^ | 0.26 |
| Ndc80 depleted | Region B^Ndc80p^ | 0.15 |
| Ndc80 depleted | Region C^Ndc80p^ | 0.0070 |
| Ndc80 depleted | Region A^Ndc80p^ B^Ndc80p^ C^Ndc80p^ | 0.78 |
| Ndc80 depleted | Region A^Ndc80p^ B^Ndc80p^ | 0.98 |
| Ndc80 depleted | Region A^Ndc80p^ C^Ndc80p^ | 0.60 |
| Ndc80 depleted | Region B^Ndc80p^ C^Ndc80p^ | 0.38 |
| Wild-type Ndc80 | Region A^Ndc80p^ | <0.001 |
| Wild-type Ndc80 | Region B^Ndc80p^ | <0.001 |
| Wild-type Ndc80 | Region C^Ndc80p^ | 0.0024 |
| Wild-type Ndc80 | Region A^Ndc80p^ B^Ndc80p^ C^Ndc80p^ | <0.001 |
| Wild-type Ndc80 | Region A^Ndc80p^ B^Ndc80p^ | <0.001 |
| Wild-type Ndc80 | Region A^Ndc80p^ C^Ndc80p^ | <0.001 |
| Wild-type Ndc80 | Region B^Ndc80p^ C^Ndc80p^ | <0.001 |
| Region A^Ndc80p^ | Region B^Ndc80p^ | 0.009 |
| Region A^Ndc80p^ | Region C^Ndc80p^ | <0.001 |
| Region A^Ndc80p^ | Region A^Ndc80p^ B^Ndc80p^ C^Ndc80p^ | 0.40 |
| Region A^Ndc80p^ | Region A^Ndc80p^ B^Ndc80p^ | 0.26 |
| Region A^Ndc80p^ | Region A^Ndc80p^ C^Ndc80p^ | 0.099 |
| Region A^Ndc80p^ | Region B^Ndc80p^ C^Ndc80p^ | 0.03 |
| Region B^Ndc80p^ | Region C^Ndc80p^ | 0.23 |
| Region B^Ndc80p^ | Region A^Ndc80p^ B^Ndc80p^ C^Ndc80p^ | 0.081 |
| Region B^Ndc80p^ | Region A^Ndc80p^ B^Ndc80p^ | 0.14 |
| Region B^Ndc80p^ | Region A^Ndc80p^ C^Ndc80p^ | 0.37 |
| Region B^Ndc80p^ | Region B^Ndc80p^ C^Ndc80p^ | 0.54 |
| Region C^Ndc80p^ | Region A^Ndc80p^ B^Ndc80p^ C^Ndc80p^ | 0.0023 |
| Region C^Ndc80p^ | Region A^Ndc80p^ B^Ndc80p^ | 0.0047 |
| Region C^Ndc80p^ | Region A^Ndc80p^ C^Ndc80p^ | 0.035 |
| Region C^Ndc80p^ | Region B^Ndc80p^ C^Ndc80p^ | 0.054 |
| Region A^Ndc80p^ B^Ndc80p^ C^Ndc80p^ | Region A^Ndc80p^ B^Ndc80p^ | 0.77 |
| Region A^Ndc80p^ B^Ndc80p^ C^Ndc80p^ | Region A^Ndc80p^ C^Ndc80p^ | 0.42 |
| Region A^Ndc80p^ B^Ndc80p^ C^Ndc80p^ | Region B^Ndc80p^ C^Ndc80p^ | 0.21 |
| Region A^Ndc80p^ B^Ndc80p^ | Region A^Ndc80p^ C^Ndc80p^ | 0.58 |
| Region A^Ndc80p^ B^Ndc80p^ | Region B^Ndc80p^ C^Ndc80p^ | 0.32 |
| Region A^Ndc80p^ C^Ndc80p^ | Region B^Ndc80p^ C^Ndc80p^ | 0.74 |
