# Supplementary material for: The Ndc80 complex bridges two Dam1 complex rings
Source: eLife. 2017 Feb 13;6:e21069. doi: 10.7554/eLife.21069 (PMC5354518; doi:10.7554/eLife.21069)
Supplement: Supplementary file 2. — DOI: http://dx.doi.org/10.7554/eLife.21069.030 [file elife-21069-supp2.docx]

A. Table of yeast strains used in this study

| Strain | Genotype^a^ | Reference |
| --- | --- | --- |
| W303 | *ade2‐1oc can1‐100 his3‐11,15 leu2‐3,112 trp1‐1 ura3‐1* |  |
| JTY5-8b | *ade3∆-100 cyh2^r^ lys2∆::HIS3 ndc80∆::natMX* [pJT12] | Tien et al., 2013 |
| JOKY3 | *ade3∆-100 cyh2^r^ lys2∆::HIS3 ndc80∆::natMX* [pJT12] [pJOK013] | This study |
| JOKY34 | *ade3∆-100 cyh2^r^ lys2∆::HIS3 ndc80∆::natMX* [pJT12] [pJT14] | This study |
| JOKY35 | *ade3∆-100 cyh2^r^ lys2∆::HIS3 ndc80∆::natMX* [pJT12] [pRS316] | This study |
| JOKY4 | *ade3∆-100 cyh2^r^ lys2∆::HIS3 ndc80∆::natMX* [pJT12] [pJOK013] [pJOK018] | This study |
| JOKY5 | *ade3∆-100 cyh2^r^ lys2∆::HIS3 ndc80∆::natMX* [pJT12] [pJOK013] [pJOK017] | This study |
| JOKY15 | *ade3∆-100 cyh2^r^ lys2∆::HIS3 ndc80∆::natMX* [pJT12] [pJOK013] [pRS315] | This study |
| JOKY16 | *ade3Δ-100 ura3-1::NDC80::URA3 CEN3::33LacO::kan pCUP1-GFP12LacI12::HIS leu2::pGPD1-OsTIR1:LEU2 SPC110-Cherry::hphMX NDC80-3V5-IAA7:kanMX* | This study |
| JOKY17 | *ade3Δ-100 ura3-1::ndc80-940::URA3 CEN3::33LacO::kan pCUP1-GFP12LacI12::HIS leu2::pGPD1-OsTIR1::LEU2 SPC110-Cherry::hphMX NDC80-3V5-IAA7::kanMX* | This study |
| JOKY18 | *ade3Δ-100 ura3-1::ndc80-1148::URA3 CEN3::33LacO::kan pCUP1-GFP12LacI12::HIS leu2::pGPD1-OsTIR1::LEU2 SPC110-Cherry::hphMX NDC80-3V5-IAA7::kanMX* | This study |
| JOKY19 | *ade3Δ-100 ura3-1::ndc80-1687::URA3 CEN3::33LacO::kan pCUP1-GFP12LacI12::HIS leu2::pGPD1-OsTIR1::LEU2 SPC110-Cherry::hphMX NDC80-3V5-IAA7::kanMX* | This study |
| JOKY20 | *ade3Δ-100 CEN3::33LacO::Kan pCUP1-GFP12LacI12::HIS leu2::pGPD1-OsTIR1::LEU2 SPC110-Cherry::hphMX NDC80-3V5-IAA7::kanMX* | This study |
| JOKY47 | *ade3∆-100 cyh2^r^ lys2∆::HIS3 ndc80∆::natMX* [pJT12] [pJOK035] [pJOK038] | This study |
| JOKY40 | *ade3∆-100 cyh2^r^ lys2∆::HIS3 ndc80∆::natMX* [pJT12] [pRS316] [pJOK038] | This study |
| JOKY48 | *ade3∆-100 cyh2^r^ lys2∆::HIS3 ndc80∆::natMX* [pJT12] [pJOK036] [pJOK038] | This study |
| JOKY51 | *ade3Δ leu2-3::pGPD1-OsTIR1::LEU2 ura3-1::URA3 SPC110-Cherry::hphMX NDC80-3V5-IAA7:kanMX DAD4-CFP::hphMX MTW1-Venus::kanMX* | This study |
| JOKY52 | *ade3Δ leu2-3::pGPD1-OsTIR1::LEU2 ura3-1::NDC80::URA3 SPC110-Cherry::hphMX NDC80-3V5-IAA7:kanMX DAD4-CFP::hphMX MTW1-Venus::kanMX* | This study |
| JOKY53 | *ade3Δ leu2-3::pGPD1-OsTIR1::LEU2 ura3-1::ndc80-A^b^::URA3 SPC110-Cherry::hphMX NDC80-3V5-IAA7:kanMX DAD4-CFP::hphMX MTW1-Venus::kanMX* | This study |
| JOKY54 | *ade3Δ leu2-3::pGPD1-OsTIR1::LEU2 ura3-1::ndc80-B^b^::URA3 SPC110-Cherry::hphMX NDC80-3V5-IAA7:kanMX DAD4-CFP::hphMX MTW1-Venus::kanMX* | This study |
| JOKY55 | *ade3Δ leu2-3::pGPD1-OsTIR1::LEU2 ura3-1::ndc80-C^b^::URA3 SPC110-Cherry::hphMX NDC80-3V5-IAA7:kanMX DAD4-CFP::hphMX MTW1-Venus::kanMX* | This study |
| JOKY56 | *ade3Δ leu2-3::pGPD1-OsTIR1::LEU2 ura3-1::ndc80-A^b^B^b^C^b^::URA3 SPC110-Cherry::hphMX NDC80-3V5-IAA7:kanMX DAD4-CFP::hphMX MTW1-Venus::kanMX* | This study |
| JOKY57 | *ade3Δ leu2-3::pGPD1-OsTIR1::LEU2 ura3-1::ndc80-A^b^B^b^::URA3 SPC110-Cherry::hphMX NDC80-3V5-IAA7:kanMX DAD4-CFP::hphMX MTW1-Venus::kanMX* | This study |
| JOKY58 | *ade3Δ leu2-3::pGPD1-OsTIR1::LEU2 ura3-1::ndc80-A^b^C^b^::URA3 SPC110-Cherry::hphMX NDC80-3V5-IAA7:kanMX DAD4-CFP::hphMX MTW1-Venus::kanMX* | This study |
| JOKY59 | *ade3Δ leu2-3::pGPD1-OsTIR1::LEU2 ura3-1::ndc80-B^b^C^b^::URA3 SPC110-Cherry::hphMX NDC80-3V5-IAA7:kanMX DAD4-CFP::hphMX MTW1-Venus::kanMX* | This study |

^a^All strains have the same markers as W303 except as noted

^b^ Indicates the region of the mutation. “A,” “B,” and “C” signify a 15 bp insertion at base pair 940, 1148, and 1687, respectively.

Table S7. plasmids used in this study

| Plasmid | Relevant markers | Reference |
| --- | --- | --- |
| pRS315 | *CEN6 ARSH4 LEU2 bla* | Sikorski and Hieter 1989 |
| pRS316 | *CEN6 ARSH4 URA3 bla* | Sikorski and Hieter 1989 |
| pJT12 | *NDC80 ADE3 LYS2* bla in 2μm vector | Tien et al, 2013 |
| pJT14 | *NDC80* in pRS316 | Tien et al, 2013 |
| pJOK13 | *ndc80^10hep^* in pRS316 | This study |
| pJOK17 | *NUF2* in pRS315 | This study |
| pJOK18 | *nuf2^10hep^* in pRS315 | This study |
| pJOK19 | *NDC80* in pRS315 | This study |
| pJOK35 | *NDC80-FLAG* in pRS316 | This study |
| pJOK36 | *ndc80^10hep^* in pRS316 | This study |
| pJOK38 | *nuf2^10hep^* in pRS315 | This study |
